# Exploring the use, benefits, and challenges of nutrition applications among clinical nutrition specialists in Saudi Arabia

**DOI:** 10.3389/fnut.2026.1766014

**Published:** 2026-07-10

**Authors:** Hala A. Alsultani, Alyaa M. Zagzoog

**Affiliations:** Department of Community Health, Faculty of Applied Medical Sciences, Northern Border University, Arar, Saudi Arabia

**Keywords:** artificial intelligence, clinical nutrition, dietitians, digital health adoption, mixed methods, nutrition applications, reflexive thematic analysis

## Abstract

**Background/Objectives:**

Technological advancements in Saudi Arabia have influenced healthcare delivery, including clinical nutrition practice. This study examined the impact of digital nutrition tools on clinical dietitians' work, focusing on perceived benefits, challenges, and experiences with technology integration.

**Methods:**

A mixed-methods design was employed. Quantitative data were collected through a self-developed online survey of senior clinical nutrition students, interns, and practicing clinical dietitians (*n* = 272). Qualitative data were obtained via semi-structured online interviews with 10 participants. Surveys assessed tool usage, perceived benefits, and challenges, while interviews explored participants' experiences in depth. Quantitative data were analyzed using descriptive statistics, and qualitative data were examined using Braun and Clarke's reflexive thematic analysis to ensure rigor, credibility, and cultural relevance.

**Results:**

Survey participants were predominantly female, Saudi nationals with bachelor's degrees in clinical nutrition and 1 to 5 years of professional experience. Respondents generally agreed on the usefulness of digital tools; however, perceived barriers differed significantly by educational level (*p* = 0.047) and professional role (*p* = 0.037), whereas perceived benefits did not differ significantly across either variable (*p* > 0.05). Qualitative interviews indicated that digital tools supported efficiency, accuracy, and patient care. Four main themes emerged: technology as a catalyst for efficiency, risks of overreliance, cultural and systemic gaps, and emerging interest in artificial intelligence (AI), including general-purpose tools and specialized clinical AI platforms for dietary assessment and food recognition, with variable participant ability to distinguish between the two. Concerns included application reliability and the need to maintain manual calculation skills.

**Conclusions:**

Digital nutrition tools can facilitate aspects of clinical practice, including workflow and patient care. However, balanced use is important to maintain professional competence and preserve critical manual skills, suggesting that technology should complement, rather than replace, core clinical expertise.

## 1 Introduction

In recent years, Saudi Arabia has made considerable progress in integrating digital technologies into healthcare, particularly in clinical nutrition (1). As demand for efficient, patient-centered services grows, digital platforms have become essential tools for dietitians, improving communication, data accessibility, and patient management (2). Telehealth services, mobile applications, and nutritional databases have transformed dietary care by enabling personalized interventions tailored to individual needs (3). Additionally, artificial intelligence (AI) and data analytics enhance the accuracy and efficiency of dietary assessments, supporting evidence-based decision-making and improved clinical outcomes (4). AI-powered food recognition technologies now assist in analyzing meal composition, reducing self-reporting errors, and improving dietary precision (5). Several studies have demonstrated that mobile applications promote adherence to nutritional goals by providing real-time feedback and tracking food intake (6, 7). Despite these advances, challenges remain in effectively integrating such technologies into routine clinical practice. This study aims to explore the impact of digital tools on the efficiency, accuracy, and overall effectiveness of clinical nutrition practice in Saudi Arabia.

Accurate calculation of calorie and macronutrient requirements is central to clinical nutrition and is traditionally performed manually using reference tables and predictive equations based on BMI, medical condition, and activity level (8). Manual methods are time-consuming, prone to error, and may lack precision in complex cases, including patients with chronic diseases or fluid imbalances. Automation and digital tools can reduce cognitive workload, improve accuracy, and streamline routine calculations, enabling dietitians to focus on individualized patient care (9). These tools also facilitate standardized documentation, data-driven decision-making, and quality improvement initiatives.

Despite their potential, adoption of digital nutrition technologies faces multiple barriers. Dietitians often experience time constraints and heavy patient loads, limiting their capacity to fully use digital tools (10). Infrastructure challenges, particularly in rural areas, such as poor internet connectivity, unreliable electricity, and limited data storage, further impede implementation (11). Data privacy and security concerns remain critical, as large-scale data collection increases the risk of breaches and misuse (12). Transparent, unbiased, and user-friendly algorithmic nutrition models are essential to ensure trust and safe clinical use (13). Accordingly, this study investigates the impact of nutrition applications on the daily clinical work of dietitians in Saudi Arabia, examining how digital tools influence efficiency, accuracy, administrative workload, and patient-centered care, providing evidence to guide effective integration of technology in clinical nutrition practice.

## 2 Materials and methods

This study utilized a mixed-methods design, integrating quantitative and qualitative approaches to examine the impact of nutrition applications on the daily clinical practice of clinical nutrition specialists. Quantitative data were collected through a survey designed to capture specialists' perspectives on the need for, prior experiences with, and perceived challenges and opportunities of using nutrition applications and related technologies. To complement this, qualitative data were obtained through in-depth semi-structured interviews, providing deeper insights into participants' experiences and perceptions. Together, this design allowed for both measurable outcomes and rich narrative accounts, offering a comprehensive understanding of how nutrition applications influence the work of clinical nutrition specialists within Saudi Arabia's healthcare system.

### 2.1 Sampling and recruitment

This study recruited three target groups from across Saudi Arabia: senior clinical nutrition students (fourth year), clinical nutrition interns, and practicing clinical dietitians. Eligible participants included:

- Fourth-year (senior) clinical nutrition students in Saudi Arabia,
- Clinical nutrition interns in Saudi Arabia, or
- Clinical dietitians practicing in Saudi Arabia.

Exclusion criteria included clinical nutrition students who were not in their senior year, clinical nutrition graduates who had never practiced, and graduates without professional certification. Senior clinical nutrition students were included as a deliberate third group to capture digital tool readiness across training stages, relevant to pre-registration curriculum design. As they lack direct clinical experience, their findings are interpreted separately from those of practicing clinicians where claims relate to workplace integration.

A mixed-methods approach was employed, combining survey distribution with follow-up interviews. To maximize reach and accessibility, the survey link was disseminated through social media platforms, including LinkedIn, WhatsApp, and X (formerly Twitter). In addition, recruitment flyers and survey links were emailed directly to the target population to enhance the response rate. The minimum required sample size was determined using G^*^Power v3.1.9.9, with an *a priori* power analysis specifying a one-tailed test, effect size *d* = 0.2, α = 0.05, and power = 0.80, yielding a recommended sample of 156 participants.

As the survey was distributed through open social media channels and institutional email lists, the total number of individuals reached could not be determined and the response rate is therefore not calculable. The achieved sample (*n* = 272) exceeded the minimum required by *a priori* power analysis (G^*^Power v3.1.9.9; *d* = 0.2; α = 0.05; power = 0.80; minimum *n* = 156), providing adequate statistical power. Participants were predominantly aged 20–25 years (50.7%), followed by 26–30 years (23.9%) and 31–39 years (22.8%).

### 2.2 Data collection

#### 2.2.1 Quantitative data

A cross-sectional survey was administered online via Microsoft Outlook. As no validated tool met the objectives of this study, the research team developed a tailored questionnaire. Content validity was assessed through expert review by clinical nutrition specialists, including degree holders and PhD-level practitioners, who evaluated clarity, relevance, and comprehensiveness. A pilot test was subsequently conducted to identify and correct errors prior to full data collection. While a formal Content Validity Index (CVI) was not calculated, these steps were taken to enhance instrument validity. The final survey began with demographic questions, followed by items addressing the use of nutrition applications (Table 1). At the conclusion, participants were invited to provide their email and phone number if they were interested in a 45–60 min virtual interview. Responses were organized, categorized, and analyzed to assess application usage, perceived benefits, and implementation challenges.

**Table 1:** Questionnaire core questions.

To what extent do you agree with the following statements? (Strongly agree–agree–neutral–disagree–strongly disagree)
The field of clinical nutrition is behind in adopting technological advancements.
Habitual reliance on manual calculation methods limits the adoption of technology in nutrition practice.
Limited availability or restricted access to technological tools is a major challenge for nutrition specialists.
Inaccurate or outdated information reduces trust in electronic nutrition platforms.
The cost of technology and nutrition applications presents a barrier to its use in nutrition practice.
Technology and nutrition applications improve the speed of determining patients' macronutrient requirements.
Technology supports nutrition experts in providing individualized nutritional care.
Technology enables nutrition experts to prioritize nutritional interventions based on laboratory results.
Using technology and nutrition applications for calculations (e.g., BMI, calorie needs, protein, macronutrients) increases clinical accuracy.
Technology for collecting and analyzing patient data enhances the quality of nutritional care.
Integrating technology into nutrition practice helps reduce workload stress.
Education and training on the use of technology are important for nutrition specialists.

#### 2.2.2 Qualitative data

In-depth semi-structured interviews were conducted with participants who volunteered to join a virtual, individual session (45–60 min) via Zoom. Purposive sampling was used to ensure diversity in professional role, gender, and geographic region across Saudi Arabia. Data saturation was achieved after 10 interviews. The interview guide included informed consent procedures, one opening question, multiple core questions (Table 2), and a closing question. At the conclusion of the interview, participants were offered a 35% discount on their subscription to NutriCare, a nutrition platform developed by Saudi clinical dietitians, as a token of appreciation for their time. This discount was obtained through a promotional arrangement with the platform provider; the authors have no financial or professional affiliation with NutriCare. With participant consent, all sessions were video-recorded using Zoom (version 5.9.1) and conducted by two researchers (H. A. A and A. M. Z).

**Table 2:** Interview core questions.

Nutritional calculations
Tools used to calculate patient/client nutritional needs. ° Perceived ease of use and user-friendliness. ° Systems or methodologies followed. Perceptions of ease and time required for calorie and macronutrient calculations. Comparison of time spent on assessments, calculations, and lab reviews vs. patient education and dietary counseling. ° Satisfaction with this balance.
Use of nutrition applications and technologies
Global and local (Saudi) applications used in clinical nutrition practice (hospitals, health centers, fitness clubs). ° Applications personally used vs. those used by colleagues. Familiarity with Saudi platforms such as: ° NutriCare (calorie, protein, and nutrient calculations). Clinical Nutrition application (enteral nutrition, formula selection, and lab interpretation). ° Perceptions of the role and impact of these applications in daily clinical nutrition practice (students, interns, and dietitians). Comparison of speed, quality, and outputs between traditional calculation methods and smart applications.
Opportunities and challenges
Perceived impact of nutrition applications on: ° Professional performance of dietitians. ° Quality of patient/client care. ° Costs and resource use (considering paid vs. free platforms). ° Time efficiency. Concerns regarding patient data privacy and security. Personal stance on the adoption of applications (from the perspective of dietitians, supervisors, professors, or preceptors). Potential research contributions to the field of nutrition and digital applications. Suggestions for innovative tools or applications to support dietitians in clinical practice.

#### 2.2.3 Ethics approval and considerations

Ethical approval for this study was obtained from the Local Committee of Bioethics (LCBE) at Northern Border University. Informed consent was obtained in two stages: first, at the beginning of the online survey, and subsequently via email, where participants reviewed the consent form and provided their signature prior to the interview. To ensure ethical rigor, each interview commenced with a confidentiality statement, and participants were reminded that the session would be audio-recorded. Although no direct risks were anticipated, the research team remained attentive to cultural, religious, and personal sensitivities, as well as participants' individual opinions and values. Confidentiality was safeguarded by securely storing all data on the research team's Northern Border University OneDrive account. Throughout the research process, efforts were made to authentically represent participants' voices while clearly distinguishing between their perspectives and the researchers' interpretations. These measures were integrated into both data collection and analysis to uphold ethical integrity.

### 2.3 Quantitative analysis approach

Data were analyzed using IBM SPSS Statistics version 26. Descriptive statistics (frequencies, means, standard deviations, quartiles) summarized participant responses. Group differences were tested with the median test (α = 0.05), a non-parametric alternative to one-way ANOVA, as Shapiro–Wilk results showed significant non-normality for both scales (*p* < 0.001). The questionnaire included two five-point Likert scales: (1) attitudes toward the benefits of technology use in clinical nutrition and (2) perceived barriers. Responses ranged from 1 (“completely disagree”) to 5 (“strongly agree”). For each participant, scores were calculated as the mean of coded responses (range 1–5). Higher scores on the benefits scale indicated more favorable attitudes, while higher barrier scores reflected stronger agreement with the presence of obstacles.

### 2.4 Qualitative analysis approach

The study aimed to conduct interviews with participants across three levels of clinical nutrition—senior students, interns, and practicing dietitians in Saudi Arabia—to enhance the depth and transferability of the qualitative findings. A semi-structured interview protocol guided the process, encompassing informed consent, introductory prompts, core questions, and closing remarks. Interviews were conducted virtually using Zoom (version 5.9.1), which minimized logistical challenges and potential distractions. Sessions were led by a researcher with oversight from A. M. Z, an expert in qualitative interviewing, to ensure methodological rigor. Zoom has been widely recognized as a reliable platform for qualitative data collection and serves as an effective alternative to in-person interviews.

#### 2.4.1 Interview analysis

Descriptive statistics, including frequencies and percentages, were calculated using Microsoft Excel (v16.98) to summarize participants' demographic characteristics. Qualitative interviews were analyzed following Braun and Clarke's (29) six-phase reflexive thematic analysis, capturing both semantic and latent meanings to reflect workplace experiences and challenges. Two researchers, H. A. A and A. M. Z, independently analyzed each transcript, incorporating peer debriefing and self-reflection to enhance analytical rigor. Interviews were conducted virtually, audio-recorded, and transcribed verbatim using Transkriptor (v3.0.4). Sample adequacy was supported by the information power model, ensuring sufficient data richness and alignment with qualitative research standards (29). Since interviews were conducted in Arabic, participant quotes were translated into English and back-translated by two bilingual researchers to ensure accuracy.

This approach promoted credibility, cultural sensitivity, and depth in interpreting participants' perspectives. Analytical memos were maintained to document decisions throughout the process. Initial themes included: “the importance of balance and not depending solely on programs,” “updating the guidelines regarding applications is important,” and “keeping up with technology and AI to be ready to receive patients.” Using Braun and Clarke's framework, H. A. A and A. M. Z conducted the first three phases independently, while A. M. Z led the reviewing, defining, and reporting of themes, ensuring a systematic and rigorous analysis. To protect participant confidentiality, all names presented in the qualitative results section are pseudonyms. Participants were informed of this procedure in the informed consent form, and this approach was approved by the institutional ethics committee.

## 3 Results

### 3.1 Quantitative approach

#### 3.1.1 Sample characteristics and demographics

A total of 272 participants met the eligibility criteria and completed the survey. Most participants were female (*n* = 232, 85.3%); Saudi nationals (*n* = 267, 98.2%); holding a BSc (*n* = 211, 77.6%) or a university degree in general (*n* = 263, 96.7%); working as clinical dietitians (*n* = 201, 73.9%), and having work experience within 10 years (*n* = 171, 63.1%). Nearly half of the participants were from the Western region of Saudi Arabia (*n* = 128, 47.1%; Table 3). As noted in the Sampling and Recruitment section, findings from senior clinical nutrition students (*n* = 31) are interpreted separately where claims relate to clinical workplace integration, as their perspectives reflect pre-clinical readiness rather than direct workplace experience.

**Table 3:** Sample demographic and characteristics.

Variable	Categories	Frequency (*N* = 272)	%
Gender	Male	40	14.7
	Female	232	85.3
Age	20–25	138	50.7
	26–30	65	23.9
	31–39	62	22.8
	40–60	6	2.2
	60+	1	0.4
Nationality	Saudi	267	98.2
	Non-Saudi	5	1.8
Region	Central	59	21.7
	Northern	35	12.9
	Southern	23	8.5
	Eastern	27	9.9
	Western	128	47.1
City	Jeddah	37	13.6
	Riyadh	44	16.2
	Arar	26	9.6
	Medina	47	17.3
	Makkah	36	13.2
	Jazan	19	7.0
	Other	63	23.2
Education level	Secondary	9	3.3
	BSc	211	77.6
	MSc	46	16.9
	PhD	6	2.2
Current job title	Clinical dietitians	201	73.9
	Interns	40	14.7
	Senior clinical nutrition students	31	11.4
Experience (years)	No experience	31	11.4
	< 1	83	30.6
	1–5	88	32.5
	6–10	47	17.3
	11–20	19	7.0
	>20	3	1.1

#### 3.1.2 Responses to the main scales

Table 4 indicates that all benefit-related items had mean scores above 4, reflecting general agreement regarding the advantages of technology in clinical nutrition. This was consistent with the overall benefits score (*M* = 4.59, *SD* = 0.46). Similarly, all barrier-related items averaged around four or higher, indicating agreement on the validity of barriers, which corresponded with a high overall barriers score (*M* = 4.17, *SD* = 0.58).

**Table 4:** The association between education / job title and the perception toward the benefits / barriers regarding the use of technology.

Variable	Categories	Score of benefits/advantages			Score of barriers		
		*M* ±*SD*	Median (IQR)	*P*-value	*M* ±*SD*	Median (IQR)	*P*-value
Education	Secondary	4.67 ± 0.42	4.71 (4.5–5)	0.326	4.48 ± 0.52	4.67 (3.92–4.92)	0.047
	BSc	4.61 ± 0.46	4.71 (4.29–5)		4.23 ± 0.53	4.17 (3.83–4.67)	
	MSc	4.54 ± 0.46	4.71 (4.14–5)		3.96 ± 0.67	4.17 (3.63–4.33)	
	PhD	4.24 ± 0.6	4.36 (3.71–4.68)		3.47 ± 0.96	3 (2.79–4.5)	
Job title	Clinical dietitians	4.59 ± 0.47	4.71 (4.29–5)	0.934	4.14 ± 0.59	4.17 (3.83–4.5)	0.037
	Clinical nutrition interns	4.59 ± 0.45	4.71 (4.14–5)		4.19 ± 0.64	4.25 (3.58–4.79)	
	Clinical nutrition students	4.59 ± 0.44	4.71 (4.29–5)		4.41 ± 0.4	4.5 (4–4.83)	

Subgroup comparisons by professional role are exploratory; findings involving the senior student group (*n* = 31) should be interpreted with caution due to the small subgroup size. Among professional groups, senior clinical nutrition students reported the highest barrier scores (*M* = 4.41, *SD* = 0.40), which should be interpreted in the context of pre-clinical readiness rather than direct workplace experience.

#### 3.1.3 Participation in the interview

More than one third of those completed the survey (35.3%, *n* = 96) agreed to participate in the virtual interviews (Supplementary Figure 1). Examination of participation rates across demographic groups indicates that males and females demonstrated similar levels of engagement, with participation rates of 27.5 and 36.6%, respectively (Supplementary Figure 2). Additionally, participation rate in the virtual interviews by various demographic groups is shown in Supplementary Table 1. Individuals holding a PhD appeared less inclined to participate (0%) compared with lower educational levels (22–37%), although this observation is limited by the small PhD sample size (Supplementary Figure 3). Clinical nutrition interns exhibited the highest participation rate (52.5%), relative to fourth-year students (19.4%) and clinical dietitians (34.3%; Supplementary Figure 4). Participation did not show a clear pattern across work experience levels, varying between 19 and 43% without a consistent increase or decrease with higher experience (Supplementary Figure 5). Overall, these findings suggest differential willingness to participate among professional and educational subgroups.

### 3.2 Qualitative approach

#### 3.2.1 Participant characteristics

Ten participants who completed the survey also participated in 45–60 min in-depth, semi-structured virtual interviews. Half of the participants (50%) were clinical dietitians, four (40%) were clinical nutrition interns, and one (10%) was a senior clinical nutrition student. The majority (70%) identified as female, while three participants (30%) identified as male. Regarding geographic distribution, most participants (*n* = 6, 60%) were from the Western region of Saudi Arabia. The remaining participants were from the Northern (*n* = 2, 20%), Central (*n* = 1, 10%), and Eastern regions (*n* = 1, 10%).

#### 3.2.2 Themes

The themes that emerged from qualitative analysis were technology as a catalyst for efficiency and accuracy in clinical nutrition, navigating the risks of technology in clinical nutrition, cultural and systemic gaps, and embracing technology and AI to enhance clinical nutrition practice.

**Theme 1. Technology as a catalyst for efficiency and accuracy in clinical nutrition**

The integration of technology and applications in nutrition practice was viewed by participants as a major facilitator of efficiency, accuracy, and patient care. Participants noted that such tools simplify calorie and diet calculations for both dietitians and patients, making the process quicker and more accessible. Turki (clinical dietitian) and Reema (clinical nutrition intern) emphasized that the time saved through applications not only enhances patient flow but also improves the quality of consultations. They explained that consultation time is reduced significantly, enabling dietitians to serve patients more efficiently in busy outpatient clinics. Participants further agreed that nutrition apps support precise calculations and streamline workflow, allowing more cases to be managed effectively. Lamees, a clinical nutrition intern, highlighted that “apps make planning clearer and work more precise, helping dietitians focus without rushing.” Similarly, Mai (intern) pointed out that these tools are especially valuable in pediatric care, as she found that applications minimize error margins compared to manual methods. Nonetheless, potential limitations were raised. Turki (clinical dietitian) cautioned that “applications provide accurate calculations quickly, but risks include device loss, system crashes, or network failure disrupting care.” Khalid, a clinical dietitian, added that:

Technology saves considerable effort and time. By implementing it effectively, we can see more patients daily while improving focus, quality, and accuracy. For example, tasks that previously took hours can now be completed in a fraction of the time, allowing us to treat more patients efficiently.

Some participants emphasized the importance of using applications in clinical nutrition for various reasons. In government hospitals, heavy patient loads make manual calculations impractical, with time constraints highlighted as a significant challenge by Reema, a clinical nutrition intern. Additionally, “technology allows dietitians to dedicate more attention to patients during sessions,” as noted by Lamees, another intern. Hattan (intern) also pointed out that technology simplifies nutritional assessments while providing secure and accessible storage for patient data. Reema further explained that many hospital systems already integrate the necessary tools, reducing the reliance on manual calculations. Reflecting on her own practice, Lamees (clinical nutrition intern) shared:

I used to rely on MD Calc, which provided quick calculations for BMI, ideal weight, and renal formulas. It was very helpful and evidence-based, though not universally used in all hospitals. Such applications can streamline calculations and support clinical decision-making efficiently.

Participants also listed specific applications they had used in their daily practice. For example, MDCalc was highlighted as a practical tool that delivers quick results and was used by Lamees (intern) to calculate glomerular filtration rate (GFR). A full list of applications and software is provided in Table 5. In summary, participants agreed that while technology cannot replace clinical expertise, it serves as a vital aid in reducing workload, enhancing accuracy, and improving the quality of patient care.

**Table 5:** Applications and software reported by clinical nutrition specialists.

Clinical and medical reference tools
MDCalc BMJ best practice UpToDate ICU gate
Nutrition and diet applications
MyFitnessPal Cronometer Carb count Food map Miran app CLN app iKFMC app
Pediatric and growth tools
PediTools (website) Growth charts app
Global and national resources
World health organization (WHO) website Saudi ministry of health website
Locally developed tools
Excel-based tools (local team) Google sheets (local team)
Professional preparation resources
Pass the dietitian exam

**Theme 2. Navigating the risks of technology in clinical nutrition**

Participants raised concerns about the risks of overreliance on applications in clinical nutrition, although they acknowledged the benefits of technology. Several participants worried that excessive dependence on apps might weaken fundamental skills in manual calculations and clinical reasoning. For example, Turki (clinical dietitian) cautioned that “technology saves time but may cause overreliance, leading to forgetting manual methods like Harris-Benedict.” Similarly, Fawaz (clinical dietitian) emphasized that “overreliance on technology risks reducing dietitians' manual calculation skills.” Concerns also extended to the quality and reliability of the applications themselves. Arwa (clinical dietitian) highlighted that “many nutrition apps lack updates, making them outdated and less useful over time, which discouraged continued use.” Lena (senior student) and Mai (clinical nutrition intern) expressed doubts about the validity of results generated by some apps, explaining that while such tools reduce effort and help organize diets, they may produce inaccurate outcomes and foster dependency. As Lena reflected:

Technology is valuable for saving time and improving calculation accuracy. However, it should not be relied on entirely because data may be outdated or incomplete. Therefore, balancing digital tools with manual verification is crucial for accurate clinical nutrition practice.

Participants voiced concerns that increasing dependence on applications could potentially reduce the professional demand for clinical dietitians and weaken core clinical skills. Fawaz, a clinical dietitian with over 5 years of experience, explained:

Excessive reliance on technology may reduce a therapeutic nutritionist's skills and knowledge. Specialists who depend heavily on applications risk losing their manual calculation abilities or critical thinking, highlighting the importance of balancing technology use with professional expertise to maintain competence.

At the same time, both Fawaz and Layla (clinical dietitians) noted that while calorie and macronutrient counting has become less central in many settings, it remains essential in intensive care units (ICUs) to prevent complications—making nutrition calculations still clinically relevant. Similarly, Lena (senior student) stressed that applications are less effective in complex cases, where manual methods allow for individualized adjustments that apps cannot always accommodate. As a senior student without direct clinical experience, Lena's perspective reflects pre-clinical readiness rather than workplace practice. Arwa (clinical dietitian) added that she prefers using only standardized, approved applications available within her hospital system, as these calculate nutrition requirements accurately, including protein, and therefore offer efficiency without compromising reliability. In conclusion, participants agreed that while technology is a valuable aid, safeguarding critical thinking and manual expertise remains essential to ensure dietitians' professional competence and adaptability in diverse clinical contexts. The adoption of nutrition applications must be guided by evidence-based principles, aligning with established scientific guidelines to ensure accuracy and safety. Clinical dietitians should critically evaluate digital tools before integration into patient care, prioritizing applications that adhere to recognized dietary guidelines and undergo rigorous validation.

**Theme 3. Opportunities and challenges of nutrition applications in Saudi Arabia**

Participants recognized the potential of nutrition applications to improve public health in Saudi Arabia, while also noting the practical challenges of developing and implementing effective tools. Most participants anticipated that nutrition applications could positively impact Saudi health if they were designed to be simple, practical, and also serve as educational tools for patients. Reema, an intern, shared:

I believe nutrition applications have a promising future and can positively affect public health in Saudi Arabia, where obesity and diabetes rates are high. Easy-to-use and effective tools allow clinical dietitians to manage patients' conditions, follow nutritional guidance, and engage their patients' treatments more consistently in the treatment plan.

In contrast, participants highlighted several challenges in designing effective nutrition applications or software. These included difficulties in securing sponsors, the costs of frequent software updates, the high expense of adding effective features, the lack of accredited Saudi applications, and subscription fees that limit adoption of reliable tools. Mai (intern) reflected on a personal challenge: “Finding partners or support for technological ideas in nutrition can be difficult.” Khalid, a clinical dietitian, further explained:

There are numerous nutrition applications available, but many require subscription fees, making it challenging for me as a specialist to test multiple apps for accuracy. Additionally, there is no Saudi application approved by an accredited authority, meaning independent testing of several apps is time-consuming and burdensome.

Overall, while participants were optimistic about the potential benefits of nutrition applications for patient education and public health, they emphasized that overcoming financial, technical, and regulatory barriers is essential for successful implementation in Saudi Arabia.

**Theme 4. Embracing technology and AI to enhance clinical nutrition practice**

Most participants emphasized the importance of integrating technology and artificial intelligence (AI) into the daily practice of clinical nutrition specialists, reflecting the digital evolution of healthcare. They highlighted that clinical dietitians must embrace these tools to remain effective, as failure to adapt could limit their ability to support patients. Layla, a clinical dietitian with more than 5 years of experience, explained:

We must remain current with technological advancements because patients increasingly interact with AI. Technology improves efficiency and patient care. Transitioning from paper-based methods to digital applications allows us to benefit patients effectively, stay relevant, and remain actively involved in modern nutritional practice.

Several clinical dietitians noted that calorie counting is now less central, as most patients' concerns relate more to habits and appetite. In such cases, they focus on promoting oral intake and lifestyle modifications rather than precise calorie tracking, which may be less relevant for long-term outcomes, as Layla shared. Fawaz (clinical dietitian) added that he is not concerned about negative effects from using technology long-term, emphasizing that the impact depends on how individuals use the tools, not the technology itself.

Overall, clinical nutrition specialists expressed the need for nutrition applications that provide accurate clinical calculations, culturally relevant foods, disease- and age-specific guidance, user-friendly design, reliable data, regular updates, and cost-effective, practical features (Table 6).

**Table 6:** Desired features in nutrition applications from clinical nutrition specialists perspectives.

Clinical calculations and medical support
Nitrogen-to-calorie ratio, osmolarity, and formula components Electrolyte corrections (hypernatremia, hyponatremia, hypertriglyceridemia, potassium conversions) Renal function measures (creatinine clearance, glomerular filtration rate [GFR]) Fluid balance calculations (including wounds, drains, and water flush requirements) Nutritional needs for specialized cases (burn patients, dialysis) Carbohydrate counting and insulin dosing Pediatric nutrition support, including growth chart integration and complex pediatric calculations
Cultural and regional relevance
Comprehensive analysis of Arabic food databases Coverage of traditional meals from the five regions of Saudi Arabia, including common Gulf dishes (e.g., bread and popular local foods) Updated and culturally relevant food exchange lists for Saudi cuisine Demand for a Saudi application officially approved by an accredited body and reflecting local food culture.
Population- and disease-specific guidance
Nutrition requirements tailored by age group and health condition Support for both type 1 and type 2 diabetes Disease-specific dietary recommendations (e.g., diabetes, dialysis, burns) Pediatric-focused modules with growth charts and nutrition accounts
User experience and accessibility
Built-in unit conversion tools Simple, user-friendly interface Applications that are easy to use without mandatory registration Flexibility in subscription pricing based on services offered
Data quality and reliability
High accuracy of stored nutritional information and clinical data Freedom from errors and incorrect results Regular updates to align with current clinical guidelines and food databases Assurance of cost-effectiveness and quality in outputs

## 4 Discussion

This mixed-methods study examined the integration of technology into the professional practice of nutritionists, highlighting its impact on efficiency, accuracy, and clinical stress reduction. Most participants (71.3%) strongly agreed that technology alleviates work-related stress, and 73.2% affirmed improvements in the precision of nutritional assessments. Nonetheless, barriers were evident: 50.7% reported limited access to technology, and 58.1% cited the lack of reliable digital platforms for rapid nutritional calculations. Education level and job title were significantly associated with perceived barriers (*p* < 0.05) but not with perceived benefits (*p* > 0.05). Small subgroup sizes, particularly among secondary and doctoral participants, may have affected these results, indicating the need for larger samples in future research. Qualitative data supported these findings, suggesting that while digital tools enhance efficiency and patient care—especially in high-demand hospital settings—concerns remain regarding potential overreliance on technology and the consequent erosion of fundamental clinical skills.

Although perceived benefits did not differ significantly by education level (*p* = 0.326) or professional role (*p* = 0.934), barriers differed significantly across both (*p* = 0.047 and *p* = 0.037, respectively). The universal agreement on the value of digital tools suggests that resistance to adoption is not primarily attitudinal. Rather, the markedly lower barrier scores among PhD-level practitioners (*M* = 3.47) compared to BSc holders (*M* = 4.23) and secondary-level participants (*M* = 4.48) suggest that barriers are largely systemic—including institutional funding constraints, limited access to accredited platforms, and inadequate organizational support—consistent with qualitative findings in which participants cited subscription costs and lack of institutionally approved tools as primary obstacles rather than personal reluctance.

Participants in this study emphasized the potential of culturally tailored and well-designed nutrition applications to enhance public health in Saudi Arabia through practicality, education, and contextual relevance. They highlighted the importance of incorporating artificial intelligence (AI) to support data analysis and clinical decision-making, consistent with the broader digital transformation of healthcare. Proposed features included nitrogen-to-calorie and osmolarity calculations, Arabic food databases, and fluid balance tracking, underscoring the need for technologically advanced yet locally adapted solutions to strengthen clinical nutrition practice. Similar applications of AI have shown promise internationally. In China, an AI-based nutritionist system for diabetes management achieved expert-level performance (F1 = 0.825) and demonstrated readiness for clinical pilot testing (14). Likewise, Papathanail et al. (15) developed an AI-based dietary assessment tool in Switzerland that accurately estimated nutrient intake among hospitalized older adults, improving efficiency and supporting patient-centered care.

Findings from this study indicated that technology in clinical nutrition is perceived as a catalyst for enhancing efficiency and accuracy, aligning with the first qualitative theme. Consistent with the systematic review, artificial intelligence (AI) approaches have demonstrated accuracy comparable to, and in some cases exceeding, human estimations of nutrient content derived from digital food imagery (16). A recent scoping review by Sosa-Holwerda et al. (17) found that most research focuses on dietary assessment interventions (e.g., Chatela et al. (18)), followed by lifestyle modification studies (e.g., Maher et al., (19)) and assessments for individuals with type 2 diabetes mellitus (e.g., Sun et al., (14)). A systematic review further reported AI applications primarily in smart and personalized nutrition (10 studies), dietary assessment (six), food recognition and tracking (four), predictive disease modeling (eight), and disease diagnosis (three) (20). Similarly, Folson et al. (21) validated the AI-based dietary assessment tool FRANI in Ghana, confirming comparable nutrient intake estimates to conventional methods.

The second theme of this study addressed the perceived risks associated with technology use in clinical nutrition. Participants emphasized the importance of “built-in error prevention” features within nutrition applications, consistent with König et al., who reported that data accuracy significantly influences application acceptance, as user-entered information can introduce errors, highlighting the need for transparent and reliable data sources (9). Participants also identified subscription fees as a major factor affecting application adoption. Similarly, König et al. (22) found that while payment occasionally motivated engagement, costs and in-app purchases generally discouraged continued use unless the application demonstrated clear value and quality. Furthermore, previous research indicated that consumers—rather than professionals—tend to discontinue nutrition applications due to reduced goal relevance, limited novelty, restricted functionality, usability issues, and unreliable databases, suggesting that sustained engagement depends on maintaining perceived usefulness and data credibility (23).

The third theme of this study addressed the challenges associated with using nutrition applications, with technical issues being a major concern. A systematic review reported that application crashes and slowed device performance were common barriers to adoption and continued use (22). Similarly, a survey of 279 Greek dietitians identified insufficient information or guidance on appropriate digital platforms as the main barrier to implementing the Nutrition Care Process (NCP), although 48.9% considered standardized digital platforms “very important” for effective NCP application (24). In line with these findings, 40.8% of participants in this study indicated that inaccurate or frequently updated information undermines clinical dietitians' confidence in digital tools. Data privacy and security were also highlighted as key concerns, consistent with König et al. (22), who found apprehension about unauthorized data use and potential third-party sharing.

The fourth theme identified from the qualitative data highlights the role of artificial intelligence (AI) in advancing clinical nutrition practice. Participants emphasized the importance of remaining current with technological progress in the AI era. Salinari et al. (6) demonstrated AI's potential to improve disease prediction, treatment, and real-time monitoring, thereby supporting personalized care and cost reduction. Participants also listed commonly used digital tools, aligning with Limketkai et al. (25), who found that mobile applications in clinical nutrition primarily target weight management (e.g., Cronometer, MyFitnessPal), diabetes care (e.g., Glucose Buddy, Dario Health), and gastrointestinal conditions (e.g., Cara Care, My Symptoms). Similarly, a recent review reported that professional applications such as Evolution Nutrition, Nutrium, and Practice Better facilitate individualized diet planning but should supplement, rather than replace, clinical expertise; persistent barriers include high complexity, cost, and technical limitations (26).

This study and prior research highlighted the transition from traditional to digital methods in calculating nutrient needs and implementing dietary interventions. Marin et al. (27) noted that while conventional medical protocols remain essential in post-surgical care, validated digital tools such as NutriMonitCare can enhance individualized nutrition management, patient adherence, and interdisciplinary communication. Participants in this study similarly emphasized the value of nutrition tools in pediatric care, citing reduced error margins compared to manual methods, though concerns were raised regarding network failures and device loss. Kamel et al. (28) reported the successful implementation of an electronic nutrition administration record (ENAR) integrated into the electronic health record, which improved documentation and supported nutrition-related research. Collectively, these findings underscore the importance of multidisciplinary collaboration to develop reliable, practice-oriented nutrition applications that facilitate comprehensive and data-driven clinical care.

### 4.1 The paradox of technology: efficiency gains and the risk of skill erosion

A notable paradox emerges from triangulating the quantitative and qualitative findings. While 71.3% of respondents agreed that technology reduces work-related stress, and participants consistently described how applications accelerate calculations and improve accuracy, the qualitative data simultaneously revealed sustained concern about the erosion of fundamental manual skills. Turki and Fawaz explicitly cautioned that overreliance on digital tools risks progressive loss of manual calculation ability and clinical reasoning—skills considered essential in complex or technology-disrupted clinical contexts. This ‘paradox of technology' has direct implications for long-term clinical competence and patient safety. If dietitians increasingly outsource core calculations to applications, their capacity to perform independently when systems fail or complex cases demand individualized judgment may gradually diminish—a concern particularly salient in ICU settings, where manual nutrition calculations remain clinically critical. These findings suggest that digital tool adoption should be accompanied by structured competency frameworks mandating maintenance of manual skills as a safety-critical professional standard. Regulatory and educational bodies should embed technology-independent assessments into registration and continuing professional development frameworks to guard against progressive deskilling.

### 4.2 Strengths and limitations

This study's primary limitation lies in its geographic focus on Saudi Arabia, which may restrict the generalizability of its findings to other regions with differing healthcare systems, technological infrastructures, or cultural contexts. Additionally, the use of a cross-sectional design and the sample size may limit the extent to which the results can be extrapolated beyond the study population. Moreover, this study did not collect data on participants' health status or socio-cultural-economic factors, which may influence technology adoption. Future research should consider these variables to better understand factors affecting nutrition application use. Additionally, recruitment via social media platforms (WhatsApp, X, and LinkedIn) likely introduced self-selection bias, as digitally active individuals may be more technologically inclined than the broader clinical nutrition workforce, potentially inflating positive attitudes toward digital tools. Future studies should incorporate offline recruitment channels to capture a more representative sample. The *a priori* power analysis was conducted for the total sample only; the senior student subgroup (*n* = 31) is relatively small and subgroup comparisons involving this group should be interpreted with caution. Future research should specifically power subgroup analyses to enable robust comparisons across professional roles.

Despite these limitations, the study possesses several notable strengths. Foremost among them is the adoption of a mixed-methods design, which integrates quantitative survey data with qualitative insights derived from semi-structured interviews. This methodological triangulation allows for a comprehensive understanding of technology use in clinical nutrition by combining measurable outcomes with in-depth personal narratives. Furthermore, the inclusion of diverse participant groups—clinical dietitians, interns, and senior students—captures a broad spectrum of experiences and technological competencies, thereby enhancing the internal validity and contextual relevance of the findings within Saudi Arabia. Another significant strength lies in the study's alignment with Saudi Arabia's Vision 2030, which prioritizes digital transformation in healthcare. By examining the role of digital tools in improving accuracy, reducing administrative burdens, and optimizing patient outcomes, this research contributes original empirical evidence to an emerging area of inquiry within the regional healthcare landscape.

### 4.3 Future directions and implications

The findings of this study offer valuable insights for hospitals and healthcare organizations in identifying the needs of clinical nutrition professionals and supporting their readiness to adopt digital tools. Given the generally positive attitudes toward technology observed among participants, targeted interventions—such as training workshops and access to efficient digital platforms—could enhance professional competency and streamline clinical workflows. Addressing common barriers, including limited time and inadequate technological resources, would further facilitate effective implementation, leading to improved patient care and a more supportive work environment. Future research should broaden its scope to include a larger and more diverse sample of healthcare professionals and institutions across Saudi Arabia to strengthen the generalizability of findings. Longitudinal and comparative studies are warranted to evaluate long-term impacts and cross-regional best practices in digital nutrition care. Moreover, exploring specific technological features, such as AI-assisted tools, and investing in digital literacy programs will be essential to ensure sustainable and meaningful integration of technology into clinical nutrition practice.

Future research and development efforts should prioritize several key areas. First, the integration of wearable devices into clinical nutrition practice represents a promising avenue. Wearable technologies capable of monitoring physiological parameters—including continuous glucose levels, basal metabolic rate, sleep patterns, and gut microbiome indicators—may enable more precise nutritional assessments and individualized meal planning. By providing real-time metabolic and lifestyle data, such devices could support dietitians in tailoring interventions and predicting metabolic disorders before clinical manifestation. Second, the development of evidence-based nutrition applications aligned with national and international guidelines should be prioritized. Regulatory bodies should establish accreditation frameworks to evaluate the accuracy and clinical validity of nutrition technologies, ensuring digital tools support evidence-based practice and promote meaningful patient outcomes. Third, future studies should examine the role of nutrition applications in preventing and managing metabolic disorders such as diabetes, obesity, and cardiovascular disease. Digital tools enabling real-time dietary adjustments may support early intervention and reduce the burden of diet-related chronic conditions. Collectively, these efforts will enhance evidence-based, personalized dietary care and advance the integration of technology into clinical nutrition practice. Finally, future studies should explore how health status and socio-economic factors influence professionals' adoption of digital nutrition tools.

## 5 Conclusions

This study examines the integration of digital nutrition applications into clinical practice in Saudi Arabia, outlining their potential benefits and existing challenges. The findings suggest that these tools may improve efficiency and accuracy in nutritional assessment and data management by automating routine calculations. They also indicate possible contributions to personalized nutrition guidance and patient education. Nonetheless, limitations related to affordability, functionality, infrastructure, and data privacy remain significant barriers to their broader use. Addressing these issues is necessary to ensure the appropriate and ethical implementation of digital technologies in clinical nutrition. Further research is warranted to refine application design, enhance system integration, and establish data security protocols. Overall, while digital tools present opportunities to support clinical nutrition practice, their adoption should be guided by evidence-based evaluation to ensure effectiveness, accessibility, and sustainability within healthcare systems.

## Data Availability

The original contributions presented in the study are included in the article/Supplementary material, further inquiries can be directed to the corresponding author.
