# Supplementary material for: Exploring the use, benefits, and challenges of nutrition applications among clinical nutrition specialists in Saudi Arabia
Source: Front Nutr. 2026 Jul 10;13:1766014. doi: 10.3389/fnut.2026.1766014 (PMC13395606; doi:10.3389/fnut.2026.1766014)
Supplement: Supplementary file 1 [file Table_1.docx]

Alsultani & Zagzoog

Exploring the Use, Benefits, and Challenges of Nutrition Applications Among Clinical Nutrition Specialists in Saudi Arabia

Manuscript Supplementary Data

Supplementary Table 1. Participation Rate (in the Zoom Interview) by Various Demographic Groups

| Variable | Categories | Total Participants in Category | Number Accepted within Category | % Accepted within Category |
| --- | --- | --- | --- | --- |
| Gender | Male | 40 | 11 | 27.5% |
|  | Female | 232 | 85 | 36.6% |
| Education | Secondary | 9 | 2 | 22.2% |
|  | BSc | 211 | 79 | 37.4% |
|  | MSc | 46 | 15 | 32.6% |
|  | PhD | 6 | 0 | 0.0% |
| Current job title | Clinical nutritionist | 201 | 69 | 34.3% |
|  | Clinical nutrition interns | 40 | 21 | 52.5% |
|  | Fourth-year clinical nutrition student | 31 | 6 | 19.4% |
| Experience (years) | No experience | 31 | 6 | 19.4% |
|  | < 1 | 83 | 37 | 44.6% |
|  | 1-5 | 88 | 27 | 30.7% |
|  | 6-10 | 47 | 20 | 42.6% |
|  | 11-20 | 19 | 6 | 31.6% |
|  | > 20 | 3 | 0 | 0.0% |


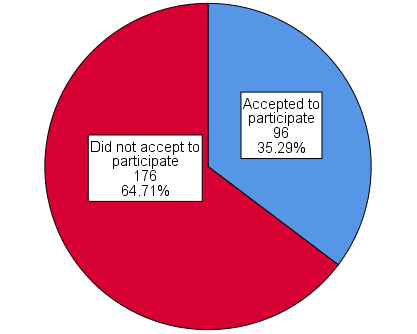


Supplementary Figure 1. Acceptance to Participate in a One-on-One Zoom Interview


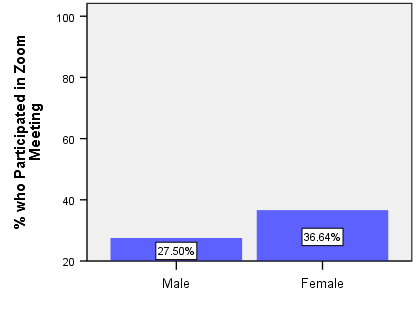


Supplementary Figure 2. Rate of Participation (in Zoom Interview) by Gender


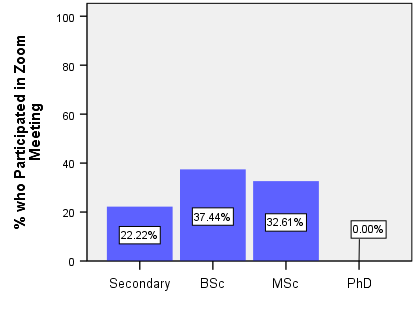


Supplementary Figure 3: Rate of Participation (in Zoom Interview) by Education


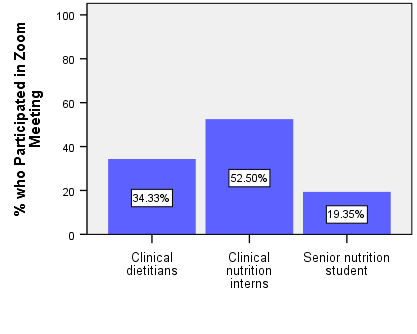


Supplementary Figure 4: Rate of Participation (in Zoom Interview) by Job Title


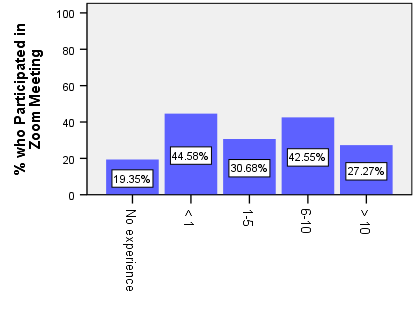


Supplementary Figure 5: Rate of Participation (in Zoom Interview) by Years of Experience
